# Need for Urticaria Guidelines in Nepal: Addressing Growing Burden of Urticaria

**DOI:** 10.31729/jnma.8948

**Published:** 2025-04-30

**Authors:** Vikash Paudel, Madhu Gyawalee, Amit Amatya, Monique Kafle, Bhaskar Mohan Meher Kayastha

**Affiliations:** 1 Department of Dermatology and Venereology, Patan Academy of Health Sciences, Lagankhel, Lalitpur, Nepal

**Keywords:** *chronic urticaria*, *dermatology*, *evidence-based practice*, *guideline development*, *health care standardization*, *quality of life*, *urticaria*

## Abstract

Urticaria is a prevalent inflammatory skin condition significantly impacting patients’ quality of life, with chronic urticaria affecting approximately 1% of the global population and 2.4% in Nepal. Despite its growing prevalence, management of urticaria in Nepal remains inconsistent due to the lack of standardized guidelines. Developing national urticaria guidelines is crucial to standardizing care, improving patient outcomes, optimizing resources, and fostering research. This process involves reviewing global best practices, adapting them to Nepal's unique healthcare context, assessing current practices, and building expert consensus. The guidelines aim to enhance diagnosis and treatment, empower patients and clinicians, and address disparities in care. Their implementation, supported by pilot testing, training, and continual updates, will ensure timely, effective, and equitable care, ultimately reducing the burden of urticaria and improving dermatological health in Nepal.

## INTRODUCTION

Urticaria, or hives, is a prevalent inflammatory skin disorder characterized by pruritus and recurrent episodes of wheals, angioedema, or both.^1^ Globally, chronic urticaria affects about 1% of the population, with acute urticaria prevalence ranging from 10-20%.^2^ A study in Nepal reported a 2.4% prevalence of urticaria..^3^ Chronic Urticaria significantly impairs patients’ quality of life, affecting both objective functioning and subjective well-being.^4-6^ Developing national urticaria guidelines is crucial to addressing the growing burden in Nepal, ensuring standardized care, improving patient outcomes, optimizing resources, and fostering research.

**Developing National Guidelines for Urticaria Management in Nepal**

The development and implementation of national urticaria guidelines in Nepal are imperative to address the growing burden of chronic urticaria. To achieve these objectives, it is imperative that the national dermatological society convenes a panel of experts to develop a consensus on urticaria management. This panel should include dermatologists, allergists, pediatrician, immunologists, pharmacologists, and other relevant stakeholders. Key experts can be identified from academic institutions like Patan Academy of Health Sciences, TU Teaching Hospital, and BPKIHS, as well as from national medical associations of dermatologist, allergologists, Nepal Medical Association, government bodies like Ministry of Health, and Nepal Health Research Council), private sector hospitals, and international collaborations. Experts should be formally invited through professional networks, and societies, with clearly defined roles. An initial national roundtable discussion should be organized, incorporating hybrid meetings to accommodate participants and assigning task forces for different guideline sections. By leveraging their collective expertise, the panel can review global guidelines, adapt them to the Nepalese context, and develop comprehensive, evidence-based national guidelines for the diagnosis, treatment, and management of chronic urticaria. These guidelines would standardize care, improve quality of life for patients, enhance clinical education, optimize resource utilization, and facilitate valuable research.^7^ By drawing on global best practices and adapting them to the local context, Nepal can ensure that all urticaria patients receive timely, effective, and equitable treatment, ultimately enhancing the overall dermatological health of the population. The outcome of national guidelines for chronic urticaria can be measured through clinical implementation audits, patient outcome tracking (using tools like Urticaria Activity Score), healthcare provider training assessments, health system impact analysis (cost-effectiveness and resource utilization), and research integration. Regular monitoring of adherence, patient quality of life, and policy adoption will ensure effectiveness and sustainability.

**The Burden of and Trends in Urticaria Globally and Regionally**

Urticaria, a widespread condition affecting millions globally, has a lifetime prevalence of chronic urticaria ranging from 0.5% to 1%.^2^ This condition significantly impacts patients’ quality of life, causing physical discomfort, emotional distress, and substantial economic burdens due to ongoing medical expenses and loss of productivity.^8^ Despite its growing prevalence, management remains inconsistent due to the absence of standardized treatment protocols and variability in drug availability. In Asia, including South Asia, the prevalence and impact of chronic urticaria are substantial, exacerbated by increasing urbanization, environmental pollution, and lifestyle changes. There is ample the need for standardized guidelines to address disparities in diagnosis, treatment, and management outcomes across the country because of high prevalence of urticaria.^3^

**The Role of Guidelines in Urticaria Management**

Many dermatological and allergy societies have worked together worldwide and have developed comprehensive guidelines for the management of urticaria, grounded in global consensus and adapted to local contexts based on drug availability and healthcare infrastructure.^1,9-10^ These guidelines serve several critical functions:

**Standardized Care:** Urticaria guidelines would provide a consistent framework for diagnosing and treating urticaria, ensuring that patients receive evidence-based, effective care.

**Education and Training:** National guidelines on urticaria can serve as educational tools for clinicians and healthcare providers, particularly in regions with limited access to specialized dermatological training. They can aid in disseminating the latest advancements and best practices in urticaria management.

**Resource Allocation:** By outlining the most effective and efficient treatment protocols, guidelines help in optimizing the use of available resources, which is particularly important in resource-constrained settings like Nepal.

**Patient Empowerment:** Clear guidelines on urticaria clinical management can also empower patients by providing them with information about their condition and the standard treatment pathways, fostering better patient engagement and adherence to treatment plans.

**Developing Urticaria Guidelines for Nepal**

The development of national urticaria guidelines in Nepal requires a systematic approach to ensure they are evidence-based, contextually relevant, and feasible for implementation. The following steps, aligned with international standard practices, outline the process:

**Reviewing Global Guidelines:** The process begins with analyzing well-established international guidelines, such as those from EAACI/GA^2^LEN/EDF/WAO.^1^ This helps identify best practices that can be adapted to Nepal's healthcare setting, ensuring the guidelines are grounded in robust evidence.

**Adapting to the Local Context:** Adapting guidelines to Nepal's unique healthcare infrastructure, patient demographics, and resource constraints is essential for practical and effective implementation. Consideration of cultural, economic, and systemic factors will enhance applicability.

**Assessment of Current Practices:** A thorough evaluation of existing management practices for chronic urticaria across healthcare settings in Nepal is critical. Identifying gaps, challenges, and variations in care will provide a foundation for the guidelines’ customization and highlight areas needing improvement.

**Consensus Building:** A multidisciplinary panel of experts, including dermatologists, allergists, pediatricians, immunologists, and public health specialists, should collaborate to adapt global guidelines. This process should consider the availability of medications, diagnostic tools, and healthcare infrastructure in Nepal. The consensus-building process will involve experts GRADE approach which will be used to evaluate evidence strength. The process includes preliminary expert meetings, literature review, multiple consensus rounds, discussion and refinement, final drafting, and pilot implementation for feedback before full adoption.

**Pilot Testing:** Before nationwide implementation, the draft guidelines should be tested in selected healthcare centers which will be decided on consensus and meeting criteria via professional networks or health authorities. Sratified sampling will be conducted to balance center types (e.g., academic vs. community).

**Nationwide Implementation:** The finalized guidelines should be introduced across the country through coordinated efforts. This includes training healthcare professionals, distributing educational materials, and establishing monitoring systems to ensure adherence.

**Continual Updates:** A mechanism should be established for periodic review and updates to the guidelines. Incorporating new evidence, emerging treatments, and feedback from implementation will keep the guidelines relevant and effective

## CONCLUSION

The increasing prevalence of urticaria in Nepal underscores the urgent need for national guidelines to standardize and improve the quality of care for this debilitating condition. By drawing on global best practices and adapting them to the local context, these guidelines can ensure that patients receive timely, effective, and equitable treatment. The collaboration between healthcare providers, policymakers, and patients is essential in this endeavor to mitigate the burden of urticaria and enhance the overall dermatological health of the population.

## References

[1] Zuberbier T, Abdul Latiff AH, Abuzakouk M, Aquilina S, Asero R (2022). The international EAACI/GA^2^LEN/EuroGuiDerm/APAAACI guideline for the definition, classification, diagnosis, and management of urticaria.. Allergy..

[2] Zuberbier T, Maurer M (2007). Urticaria: current opinions about etiology, diagnosis and therapy.. Acta Dermato-Ve-nereologica..

[3] Shrestha DP, Gurung D, Rosdahl I (2012). Prevalence of skin diseases and impact on quality of life in hilly region of Nepal.. Journal of Institute of Medicine..

[4] Gyawalee M, Paudel V (2023). Sociodemographic and clinical characteristics of chronic urticaria among patients attending the dermatology clinic in a tertiary-care hospital.. Our Dermatol Online..

[5] Paudel S, Parajuli N, Sharma RP, Dahal S, Paudel S (2020). Chronic Urticaria and Its Impact on the Quality of Life of Nepalese Patients.. Dermatol Res Pract..

[6] Das AK, Paudel U, Brar N, Karn A (2020). Impact of chronic urticaria on quality of life in a tertiary level hospital in Nepal.. Nepal Journal of Dermatology, Venereology and Leprology.

[7] Godse KV, Zawar V, Krupashankar D, Girdhar M, Kandhari S, Dhar S, Ghosh S, Rajagopalan M, Zuberbier T (2011). Consensus statement on the management of urticaria.. Indian J Dermatol..

[8] Xing Y, Wong GW (2022). Environmental Influences and Allergic Diseases in the Asia-Pacific Region: What Will Happen in Next 30 Years?. Allergy Asthma Immunol Res..

[9] Godse K, Patil A, De A, Sharma N, Rajagopalan M, Shah B, Tahiliani S, Girdhar M, Zawar V, Sangolli P, Shankar DK, Dhar S (2022). Diagnosis and Management of Urticaria in Indian Settings: Skin Allergy Research Society's Guideline-2022.. Indian J Dermatol..

[10] Song WJ, Choi M, Lee DH, Kwon JW, Kim GW, Kim MH (2020). The KAAACI/KDA evidence-based practice guidelines for chronic spontaneous urticaria in Korean adults and children: Part 1. Definition, methodology and first-line management.. Allergy Asthma Immunol Res..

